# Too Exhausted to Perform at the Highest Level? On the Importance of Self-control Strength in Educational Settings

**DOI:** 10.3389/fpsyg.2017.01290

**Published:** 2017-07-25

**Authors:** Chris Englert, Alafia Zavery, Alex Bertrams

**Affiliations:** Department of Educational Psychology, Institute of Educational Science, University of Bern Bern, Switzerland

**Keywords:** education, ego depletion, self-control, self-regulation, willpower

## Abstract

In order to perform at the highest level in educational settings (e.g., students in testing situations), individuals often have to control their impulses or desires (e.g., to study for an upcoming test or to prepare a course instead of spending time with the peer group). Previous research suggests that the ability to exert self-control is an important predictor of performance and behavior in educational contexts. According to the strength model, all self-control acts are based on one global energy pool whose capacity is assumed to be limited. After having performed a first act of self-control, this resource can become temporarily depleted which negatively affects subsequent self-control. In such a state of ego depletion, individuals tend to display impaired concentration and academic performance, fail to meet academic deadlines, or even disengage from their duties. In this mini-review, we report recent studies on ego depletion which have focused on children as well as adults in educational settings, derive practical implications for how to improve self-control strength in the realm of education and instruction, and discuss limitations regarding the assumptions of the strength model of self-control.

## Introduction

Self-control describes the process of voluntarily regulating or overriding predominant behavioral tendencies or impulses, meaning that an individual tries to resist situational urges or desires with the aim of achieving even more gratifying (long-term) goals (Baumeister et al., 1994). In educational settings, self-control seems to be particularly important (Duckworth et al., 2014). For instance, a student has to ignore irrelevant stimuli during an academic examination (e.g., noise, interfering thoughts) and to shift his/her attention on the task at hand instead (e.g., Schmeichel, 2007). Self-control is also required to deal effectively with stress (Oaten and Cheng, 2005) or to handle test anxiety (Bertrams et al., 2016). Although controlling oneself seems beneficial, previous researchers have pointed out reliably that self-control cannot always be performed successfully (for a meta-analysis, see Hagger et al., 2010).

According to the strength model of self-control, impaired self-control performance is caused by a temporary depletion of a self-control resource or strength (Baumeister et al., 1998). Baumeister et al. (1998) view self-control strength as a **metaphorical** volitional resource. It is assumed that this strength energizes all types of self-control, for instance, attention regulation (Schmeichel and Baumeister, 2010), emotion regulation (Muraven et al., 1998), or persistence (Englert and Wolff, 2015). However, the capacity of this strength is limited: Like a human muscle, one’s self-control resource can become temporarily depleted after having performed a self-control act (i.e., *ego depletion*), meaning that in a given situation an individual may not have sufficiently available self-control resources (i.e., state self-control strength) to deal with the respective self-control demands (Baumeister et al., 2007). Under ego depletion, self-control acts are performed less effectively than in situations in which one’s self-control strength is intact (cf., Hagger et al., 2010). At first sight, the strength model appears to be similar to cognitive load theories, but while cognitive load theories assume that executing two tasks **at the same time** may negatively affect performance (e.g., Sweller, 1988), the strength model postulates performance impairments in a secondary self-control task **after** having worked on a primary self-control task, meaning that there is a carry-over effect. Sticking to the strength metaphor, there may also be stable inter-individual differences in one’s general ability to control oneself, meaning that some individuals in general are more adept at regulating their impulses than others (i.e., trait self-control; Tangney et al., 2004). Previous research has repeatedly demonstrated that higher levels of trait self-control strength are positively related to several beneficial performance- and health-related variables (Tangney et al., 2004; Moffitt et al., 2011).

The effects of ego depletion on performance can be tested by adopting the two-task paradigm (Baumeister et al., 1998). In this paradigm, participants are randomly assigned to either a depletion condition or a non-depletion condition. Both groups then work a similar primary task (e.g., transcribing a neutral text by hand on a separate sheet of paper; Bertrams et al., 2010) that requires self-control in the depletion condition (e.g., omitting certain letters while transcribing the text) but does not require any self-control in the non-depletion condition (e.g., transcribing the text conventionally without any restrictions). Then, a subsequent secondary task, which is identical for both conditions, requires self-control from all participants (e.g., resisting tempting snacks; Vohs and Heatherton, 2000). It has been reliably found that in the depletion condition performance in the secondary self-control task is significantly worse than in the non-depletion condition, supporting the premise that one’s self-control strength can become temporarily depleted after one has exerted self-control in a primary task (Hagger et al., 2010).

The aim of this mini-review is to highlight the importance of state and trait self-control strength in educational settings. Furthermore, we suggest ways to improve self-control performance. Finally, we discuss limitations of the strength model of self-control.

## Empirical Findings for Self-Control Strength in Educational Settings

### Self-control Strength and Cognitive Performance

Masten and Coatsworth (1998) point out that not only cognitive abilities are important predictors of academic success but that other factors may also play a decisive role (e.g., motivation, beliefs). We agree that underperformance on academic examinations does not necessarily have to be the consequence of lower cognitive abilities (cf., Zeidner, 1998), as we argue that self-control strength is an important predictor of scholastic performance. In one of the first longitudinal studies that outlined the importance of self-control for academic performance, Mischel et al. (1988) found that children who, in general, were more adept at regulating their impulses and desires (*delay of gratification*) went on to become adults with higher scores in the SAT (see also Tangney et al., 2004; Moffitt et al., 2011).

One of the first experimental studies that investigated the effects of ego depletion on cognitive performance was conducted by Schmeichel et al. (2003). In Experiment 1 of that study, ego-depleted undergraduate students performed significantly worse than non-depleted participants on a standardized test to measure general cognitive ability that required cognitive control (Kuncel et al., 2001). Interestingly, in Experiment 2 ego depletion did not affect performance on a task that required simple cognitive operations (e.g., simple arithmetic operations). Schmeichel and colleagues argued that self-control is relevant only in tasks that require more complex cognitive operations (e.g., sustained attention) which is also in line with later research (Schmeichel, 2007; Schmeichel and Baumeister, 2010).

In another study conducted by Schmeichel et al. (2006), ego-depleted undergraduate students performed statistically significantly worse on tasks based on cognitive fluency. These tasks also required complex cognitive operations, as the individuals needed to generate novel, task-relevant thoughts (e.g., generating as many words as possible that begin with a certain letter within a certain amount of time) while at the same time they had to inhibit task-irrelevant thoughts (Benton et al., 1994). These results were confirmed by a study on creativity, in which teachers rated the performance of ego-depleted middle-school students as less creative than the performance of non-depleted students (Price and Yates, 2015).

A recent study by Englert and Bertrams (2017) confirmed Schmeichel et al.’s (2003, 2006) findings: In the Englert and Bertrams study, after manipulating self-control strength, secondary school students were instructed to memorize novel pieces of information regarding the anatomy of the human eye for 5 min. After a 1-min distraction task that consisted of simple arithmetic problems, the students were asked to recall as many pieces of information as possible. The ego-depleted participants performed significantly worse in the knowledge retrieval task than non-depleted participants. In line with Schmeichel et al. (2003), the ego-depleted and non-depleted students’ performance on the simple arithmetic problems of the distraction task did not differ, delivering additional empirical support that ego depletion seems to mainly impair more complex cognitive operations. These findings were was also documented in a recent study that showed impaired rule-defined (in contrast to less complex non-rule-defined) category learning in depleted compared with non-depleted university students (Minda and Rabi, 2015).

At the trait level, Tangney et al. (2004) found a statistically significant relationship between trait self-control and grade point average (GPA). College students with higher trait self-control had a higher GPA than students with lower levels of self-control (see also Bertrams and Dickhäuser, 2009). Duckworth and Seligman (2005) also found support for the importance of general self-control abilities, as in their longitudinal study self-control was a better predictor of academic performance than the high-school students’ IQ scores. To conclude, state self-control strength and trait self-control seem to be important antecedents for a successful academic career.

### Self-control Strength and Anxiety-Related Performance Impairments

Academic testing situations are often perceived as threatening, because successful performance on these exams is usually highly important (Zeidner, 1998). Therefore, testing situations are often associated with increased levels of anxiety (Beilock, 2008). While some studies reported a negative relationship between anxiety and cognitive performance (Beilock, 2008), other studies failed to find a reliable link between anxiety and cognitive performance (Seipp, 1991). According to attentional control theory (ACT; Eysenck et al., 2007), anxious individuals tend to ruminate about their performance in a given testing situation and are less capable of focusing their attention on the cognitive task at hand (i.e., cognitive interference), which may impair cognitive performance (Eysenck, 1992). However, Eysenck et al. (2007) argued that negative anxiety effects on attention regulation can be counteracted, by investing additional effort or by initiating certain self-regulatory processes. However, the authors did not specify the self-regulatory processes they were referring to and why individuals are not always capable to compensate for anxiety-related performance impairments. The strength model of self-control may help to bridge this conceptual theoretical gap and may also explain the inconsistent findings for the anxiety–performance relationship (see also Englert and Bertrams, 2015). Selectively controlling one’s attention can be understood as a self-control act, as task-irrelevant information needs to be voluntarily ignored, and attention needs to be focused on the task-relevant stimuli instead (Schmeichel and Baumeister, 2010). This leads to the conclusion that automatic anxiety-related attention impairments can be voluntarily overridden if an individual’s self-control strength is intact. On the contrary, in a state of ego depletion anxious individuals should be less adept at voluntarily counteracting their increased distractibility. In the former case (in a state with temporary self-control strength), there should be no effect of anxiety on performance, while in the latter case (in a state of ego depletion), there should be a negative anxiety–performance relationship. A series of studies by Bertrams and colleagues lent support for this theoretical assumption, as ego depletion moderated the anxiety–performance relationship in a broad variety of cognitive tasks (Bertrams et al., 2013, 2016; Bertrams and Englert, 2014; Englert and Bertrams, 2016).

For instance, Bertrams et al. (2013) reported that there was no statistically significant main effect of anxiety on performance on a verbal learning task (Study 1) or on a task requiring mental arithmetic operations (Study 2). Instead, anxiety had a negative effect only on performance in university students with depleted self-control strength, while the performance of participants with temporarily available self-control was not impaired under anxiety. In the same vein, secondary school students’ anxiety was negatively related to performance only on a knowledge retrieval task for students with temporarily depleted self-control strength (Bertrams and Englert, 2014).

Apart from the state component of self-control strength, it has also been demonstrated that trait self-control may play an important role in the anxiety–performance relationship (Bertrams et al., 2016). For instance, in a study by Tangney et al. (2004), higher levels of trait self-control were related to lower levels of trait anxiety in college students. These findings lead to the conclusion that state and trait self-control strength may buffer the negative effects of anxiety on attention regulation and subsequent cognitive performance. Therefore, self-control strength may be the actual self-regulatory process which had not been specified in ACT (Eysenck et al., 2007).

### Self-control Strength and Health-Related Variables

The ability to control oneself is positively associated with not only performance measures but also several health-related parameters. For instance, higher levels of self-reported state self-control strength have been found to be associated with lower levels of stress, depressive symptoms, and anxiety, indicating that state self-control strength is required to successfully deal with stressful life events and negative emotions (Ciarocco et al., 2007, Unpublished; Bertrams et al., 2011). A study by Oaten and Cheng (2005) also underlines the importance of self-control in order to successfully cope with stress, as university students’ self-control performance was less efficient during their exam period, while they also displayed a less healthy lifestyle during their exam period compared to the beginning of the semester (i.e., more junk food, less physical exercise; see also Englert and Rummel, 2016).

On the trait level, Moffitt et al. (2011) showed in a longitudinal study that childhood self-control predicted physical and mental health, as well as substance abuse in adulthood. Tangney et al. (2004) study also revealed beneficial relationships between trait self-control and several indicators of psychological adjustment as specified in the Diagnostic and Statistical Manual of Mental Disorders (DSM-IV; American Psychiatric Association, 1994), as, for example, higher levels of trait self-control were negatively related to depression, antisocial behavior, or anxiety in adults (see also Bertrams and Dickhäuser, 2009). Trait self-control has also been linked to burnout, meaning that individuals who were less adept at regulating themselves displayed higher scores on a burnout measure (Seibert et al., 2016). Finally, a study by Wirth et al. (2015) suggested that children who were diagnosed with attention deficit hyperactivity disorder (ADHD) reported lower levels of trait self-control. Furthermore, in that study the negative effects of ADHD on cognitive performance were fully mediated by trait self-control, which led the authors to conclude that performance impairments in children with ADHD were primarily caused by an impaired ability to voluntarily regulate their impulses. To conclude, high levels of state self-control strength are required to deal with stressful events and negative emotions and help to be physically active on a regular basis. Additionally, high levels of trait self-control are significantly related to a wide variety of positive health outcomes.

## Possible Interventions

In previous sections, the positive effects of self-control on behavior and health were highlighted. These findings raise the question whether there are ways to improve one’s self-control strength in educational settings. The answer to this question is yes (for an earlier overview, see Baumeister et al., 2006).

For instance, regularly performing self-control acts improves one’s self-control strength in the long run. Bray et al. (2014) asked participants to regularly perform a straining physical exercise requiring self-control over a 2-week period. At follow-up, the participants’ self-control performance was statistically significantly better compared with their baseline performance (see also Muraven, 2010). The authors explained the findings by comparing self-control strength to a human muscle, arguing that the capacity of an individual’s self-control strength can be improved by regular training, just like a human muscle. As previously mentioned, it is assumed that all acts of self-control are energized by the same limited resource (Baumeister et al., 1998), which leads to the conclusion that practicing physical self-control might also have a beneficial long-term effect on performance in cognitive self-control tasks. However, this assumption has not been tested thus far. Exercises like the one Bray et al. (2014) used could easily be integrated in physical education lessons or as breaks during other lessons in other subjects.

According to the strength model of self-control, impaired self-control performance is caused by a temporary depletion of a self-control resource or strength (Baumeister et al., 1998). It is assumed that this strength energizes all types of self-control, for instance, attention regulation (Schmeichel and Baumeister, 2010), emotion regulation (Muraven et al., 1998), or persistence (Englert and Wolff, 2015).

Studies also point to ways the educational environment can facilitate regaining depleted self-control capacities or even prevent states of ego depletion. For example, researchers have shown that granting university students a short period of rest after a primary self-control act may lead to the replenishment of depleted self-control strength (Tyler and Burns, 2008). These results can also be explained by adopting the muscle metaphor, as a human muscle needs recovery time before being fully functional again. These findings deliver a hint that it may be reasonable to grant students short breaks during lessons or before an important exam, in order to enable their self-control strength to fully recover. A recent study by Steinborn and Huestegge (2016) also found evidence for the beneficial effects of short breaks on performance in a sample of adult participants.

Furthermore, increasing students’ level of autonomy may also be a fruitful strategy to prevent performance impairments caused by low levels of self-control strength (Moller et al., 2006; Muraven et al., 2008; Muraven, 2010). If individuals have the impression that it is their decision whether they want to work on a given task or not, these tasks require less self-control strength, as the characteristic of the respective task is perceived as being less aversive. This means that less self-control strength needs to be invested to work on these tasks compared with a task that an individual is *forced* to work on (Muraven, 2010). These findings could be transferred to educational settings by offering students the opportunity to choose to some degree when and how to approach specific learning content. Students may also work on a project of their own interest. Doing something that is experienced as interesting can replenish depleted self-regulatory resources, even if the task is complex and difficult (Thoman et al., 2011).

## General Discussion

State and trait self-control strength play an important role in educational settings. In the present paper, we presented evidence that the ability to regulate certain behavioral tendencies or impulses is related to cognitive performance (Schmeichel et al., 2003), to performance in testing situations (Englert and Bertrams, 2015), as well as to several health-related variables (Tangney et al., 2004). We further demonstrated that self-control strength can be trained and systematically replenished, enabling better self-control performance (Baumeister et al., 2006; Tyler and Burns, 2008).

We would also like to discuss a recent empirical study that was not in line with the strength model of self-control. The Registered Replication Report (RRR; Hagger et al., 2016) failed to find supporting evidence for Baumeister et al.’s (1994, 1998, 2006) central assumption that working on a primary self-control task negatively affects performance on a secondary self-control task. Although the RRR has been criticized on theoretical and methodological levels (Baumeister and Vohs, 2016) and given that a previous meta-analysis supported the strength model of self-control (Hagger et al., 2010), future research endeavors should nonetheless focus on conducting additional replication studies to explain this inconsistent data. However, the published studies on self-control strength in educational settings have supported the strength model indicating that it seems highly important to prevent states of ego depletion and to increase self-control strength in order to help individuals to perform to their highest levels in educational settings.

## Author Contributions

CE, AZ, and AB substantially contributed to the writing of the manuscript. All authors approve the final version of the manuscript. The authors agree to be accountable for all aspects of the work in ensuring that questions related to the accuracy or integrity of any part of the work are appropriately investigated and resolved.

## Conflict of Interest Statement

The authors declare that the research was conducted in the absence of any commercial or financial relationships that could be construed as a potential conflict of interest.
